# Myostatin as a plausible biomarker for early stage of sarcopenic obesity

**DOI:** 10.1038/s41598-024-79534-5

**Published:** 2024-11-19

**Authors:** Chisaki Ishibashi, Kaori Nakanishi, Makoto Nishida, Haruki Shinomiya, Maki Shinzawa, Daisuke Kanayama, Ryohei Yamamoto, Takashi Kudo, Izumi Nagatomo, Keiko Yamauchi-Takihara

**Affiliations:** Health Care Division, Health and Counseling Center, Osaka University, 1-17 Machikaneyama, Toyonaka, Osaka 560-0043 Japan

**Keywords:** Myostatin, Sarcopenia, Obesity, Metabolic diseases, Biomarkers, Endocrinology

## Abstract

Since sarcopenic obesity (SO) impacts negatively on our health, early detection of SO is essential. However, prevalence of SO in an apparently healthy population has not been well examined. This study aimed to elucidate the prevalence and related factors of SO in middle-aged women, and to investigate useful diagnostic criteria for SO. Body component analyses were conducted on 432 female Osaka University employees aged 30–59 during their health checkups. Healthy (H) and SO groups were defined using cutoff values of 5.7 kg/m^2^ for skeletal muscle mass index and 30% for percent body fat. Serum myostatin and insulin levels were additionally measured. Among 432 participants, the prevalence of SO was 6.3%. Grip strength (*P* < 0.0001) was lower and triglyceride (*P* = 0.0004) and low-density lipoprotein cholesterol (*P* = 0.0105) levels, and Homeostatic Model Assessment of Insulin Resistance (*P* = 0.0262) were higher in the SO group than in the H group. Serum myostatin levels in the SO group were lower than in the H group (3,107 pg/mL vs. 3,957 pg/mL, *P* = 0.0003). Myostatin levels may be suppressed in individuals with SO without any pre-existing conditions. Our diagnostic criteria for SO could reveal the risks for metabolic-related diseases and may be useful for the early detection of SO.

**Supplementary Information:**

The online version contains supplementary material available at 10.1038/s41598-024-79534-5.

## Introduction

Sarcopenic obesity (SO) is characterized by a concomitant reduction in skeletal muscle mass and increase in body fat. Since previous studies have shown that patients with SO are more likely to have hypertension^1^, dyslipidemia^2^, metabolic syndrome^3,4^, and vascular events^5^ than those with sarcopenia or obesity alone, SO has been gaining attention in recent years. While often reported in older adults and even young to middle-aged women, those with SO have higher complication rates of metabolic syndrome^6^, type 2 diabetes^7^, and hypertension^7^ than those without SO. Moreover, SO is associated with reduced physical function in middle-aged women^8^. Thus, early detection and management of SO in the early stages is important. However, because SO is typically unaccompanied by obesity or thinness, distinguishing individuals with SO from healthy individuals is challenging. In general, SO is defined using body composition analyses. However, because body composition analyses are not usually performed during periodic health checkups at workplaces, the prevalence of SO and its related factors in relatively young populations remain unknown.

Our study also focused on myostatin, cytokine within the TGF-β family in 1997^9^. Myostatin is predominantly produced and secreted in the skeletal muscle, so serum myostatin levels are positively correlated with skeletal muscle mass^10–12^, grip strength^10,13^, lower limb muscle strength^14^, and gait speed^15^. Myostatin suppresses skeletal muscle proliferation by binding to receptors on the surface of skeletal muscle cells. Circulating myostatin levels have also been reported to be inappropriately elevated in older adults^16,17^ and patients with liver cirrhosis^18–20^, renal failure^21–23^, heart failure^24–26^ and diabetes mellitus^27^. In addition, myostatin has been implicated in visceral fat augmentation. Myostatin-knockout mice display reduced visceral fat accumulation, improved glucose tolerance^28^, and increased insulin sensitivity^29^. Myostatin signaling regulates muscle differentiation and induces insulin resistance by suppressing downstream insulin signaling^30,31^. Serum myostatin increases in obesity, positively correlates with insulin resistance indices^32,33^, and serves as a significant predictor of fat accumulation in non-obese patients with non-alcoholic fatty liver disease^34^. Myostatin is associated with skeletal muscle homeostasis in men and sarcopenia in women^17^. These findings suggest that serum myostatin may serve as a biomarker of SO, particularly in women. However, no previous studies have examined the association among myostatin, skeletal muscle reduction, and obesity in an apparently healthy population.

This study aimed to explore the prevalence of SO and its associated factors, including serum myostatin levels, in a middle-aged female population.

## Methods

### Study participants

University employees who participated in annual health examinations at Osaka University Health and Counseling Center between May 2022 and October 2022 were enrolled in this study. The inclusion and exclusion criteria for this study were as follows: (1) female individuals, (2) age between 30 and 59 years old, and (3) individuals without an acute disease on the day of their health checkups. Written informed consent was obtained from all 432 individuals. We classified the participants into four distinct groups: healthy (H), sarcopenia-only (S), obesity-only (O), and sarcopenic obesity (SO), as described in a subsequent section. Serum myostatin and insulin levels were measured in 80 participants: 53 randomly selected participants from the H group and 27 participants from the SO group.

### Clinical parameters

Clinical parameters, including age, height, body weight, body mass index (BMI), waist circumference (WC), systolic blood pressure (SBP), diastolic blood pressure (DBP), white blood cell count (WBC), hemoglobin (Hb), platelet count (Plt), aspartate aminotransferase (AST), alanine aminotransferase (ALT), γ-glutamyl transpeptidase (γGTP), blood urea nitrogen (BUN), creatinine (Cr), uric acid (UA), total cholesterol (T-Cho), triglycerides (TG), high-density lipoprotein cholesterol (HDL-C), low-density lipoprotein cholesterol (LDL-C), glucose (Glu), hemoglobin A1c (HbA1c), and exercise-related behaviors were extracted from health checkup records. Exercise-related behaviors included the frequency of engaging in sweat-inducing exercise in excess of 30 min per week, daily duration of physical activity equivalent to walking, walking speed compared with other females of approximately the same age, and daily TV/video viewing time on weekdays.

Percent body fat (PBF) and skeletal muscle mass index (SMI) were determined using the InBody 270 Body Composition Analyzer (InBody Japan, Tokyo). Grip strength was measured using a Smedley hand dynamometer (TKK5401; Takei Scientific Instruments Co., Ltd., Niigata, Japan). The finger ring test was performed by instructing participants to make a ring with the thumb and index finger of both hands around the thickest part of calf of non-dominant leg, and checking whether the calf circumference is “bigger,” “just fits” or “smaller” compared with the finger-ring circumference^35^.

Serum concentrations of myostatin and insulin were quantified using Enzyme-linked Immunosorbent Assay (GDF-8/Myostatin Quantikine ELISA Kit, R&D Systems, Minneapolis, MN, USA) and chemiluminescent enzyme immunoassay (Lumipulse Presto insulin, Fujirebio, Tokyo, Japan), respectively, using excess serum from health checkups. The serum myostatin levels were measured in duplicate and the coefficient of variation between measurements was 4.6 (2.7–6.6) %. The waist-to-height ratio (WHtR) was calculated as WC/height, and body shape index (ABSI) as WC/ (BMI^2/3 × height^1/2) [m^11/6 kg^(-2/3)]^36^. The Homeostatic Model Assessment of Insulin Resistance (HOMA-R) score was calculated as Glu (mmol/L) * insulin (µU/mL)/22.5^37^.

### Definition of SO

We defined sarcopenia as skeletal muscle mass loss indicated with SMI <  5.7 kg/m^2^, as described by the Asian Working Group for Sarcopenia in 2019^38^. Obesity was defined as having a percentage of body fat (PBF) ≥ 0%, according to the previous studies^39,40^. Subsequently, participants were classified into four groups: those with SMI ≥ 5.7 kg/m^2^ and PBF < 30% were classified into the H group, those with SMI < 5.7 kg/m^2^ and PBF < 30% into the S group, those with SMI ≥ 5.7 kg/m^2^ and PBF ≥ 30% into the O group, and those with SMI  < 7 kg/m^2^ and PBF ≥ 30% into the SO group.

### Statistical analyses

All statistical analyses were carried out using JMP® Pro 17 software (SAS Institute Inc., Cary, NC, USA). Continuous variables are expressed as median (interquartile range). The distribution of continuous variables was tested by Shapiro-Wilk test and most variables were non-normally distributed. Therefore, the Wilcoxon test was used to compare differences of continuous variables between the two groups, and the Kruskal–Wallis test followed by the post hoc Steel–Dwass test was used for multi-group comparison. The chi-square test was used to compare the proportions of categorical values. For the comparison of myostatin level between the groups, the analysis of covariate (ANCOVA) was used and adjusted for relevant factors. Relationships among clinical parameters was assessed using the Pearson’s correlation coefficient analysis. The multiple regression analysis was performed to identify independent factors for myostatin level. Explanatory variables were determined using the stepwise method. In the Pearson’s correlation coefficient analysis, the multiple regression analysis, and the ANCOVA, non-normally distributed variables were log-transformed. Statistical significance was set at *P* < 0.05.

### Study approval

The research protocol was approved by the Ethics Committee of the Health and Counseling Center of Osaka University and adhered to the principles outlined in the Declaration of Helsinki. All patients provided written informed consent before participation.

## Results

### Clinical characteristics of SO

**Fig. 1:**
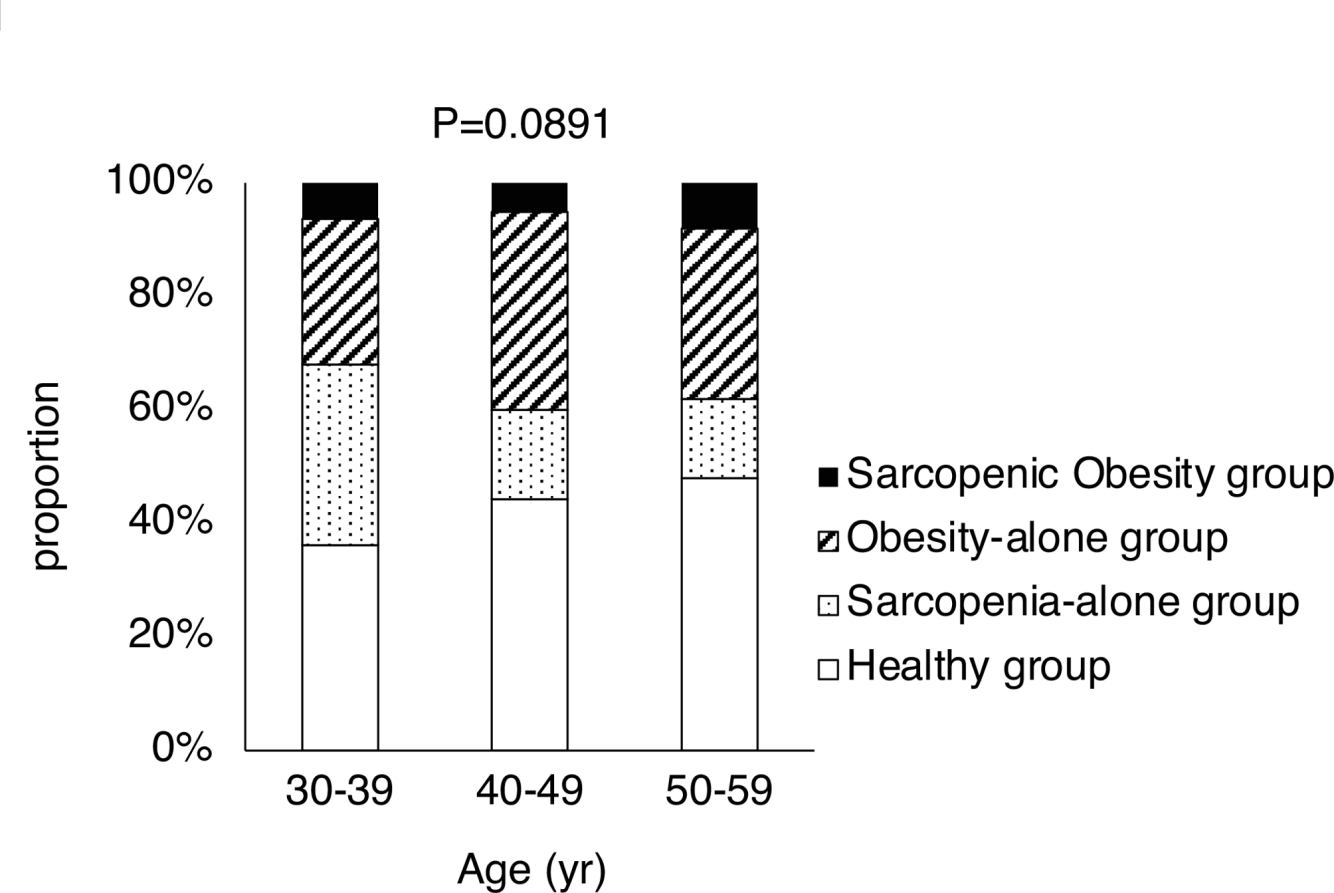
Proportion of participants classified into the healthy, sarcopenia-only, obesity-only, and sarcopenic obesity groups. Data are presented as the proportion of participants in each group according to age bracket. The chi-square test was used to compare proportions among the age groups. Age 30–39: *n* = 47, age 40–49: *n* = 235, age 50–59: *n* = 150.

Of the 432 participants, 193 were classified into the healthy (H) group, 73 into the sarcopenia-only (S) group, 139 into the obesity-only (O) group, and 27 into the SO group. Supplementary Table S1 provides the detailed clinical background information for each group. The overall prevalence of SO in the study population was 6.3% (27 out of 432). The proportions of groups in each generation are shown in Fig. 1. No significant differences were observed among each generation (*P* = 0.0891). The prevalence rates of SO were 6.4% for individuals in their 30s (*n* = 47), 5.1% for those in their 40s (*n* = 235), and 8.0% for those in their 50s (*n* = 150). While the prevalence rates of S were significantly different (*P* = 0.0131), a significant difference was not observed regarding the prevalence rates of SO among each generation (*P* = 0.5195).

### Relationship between SO and exercise-related behaviors

**Fig. 2:**
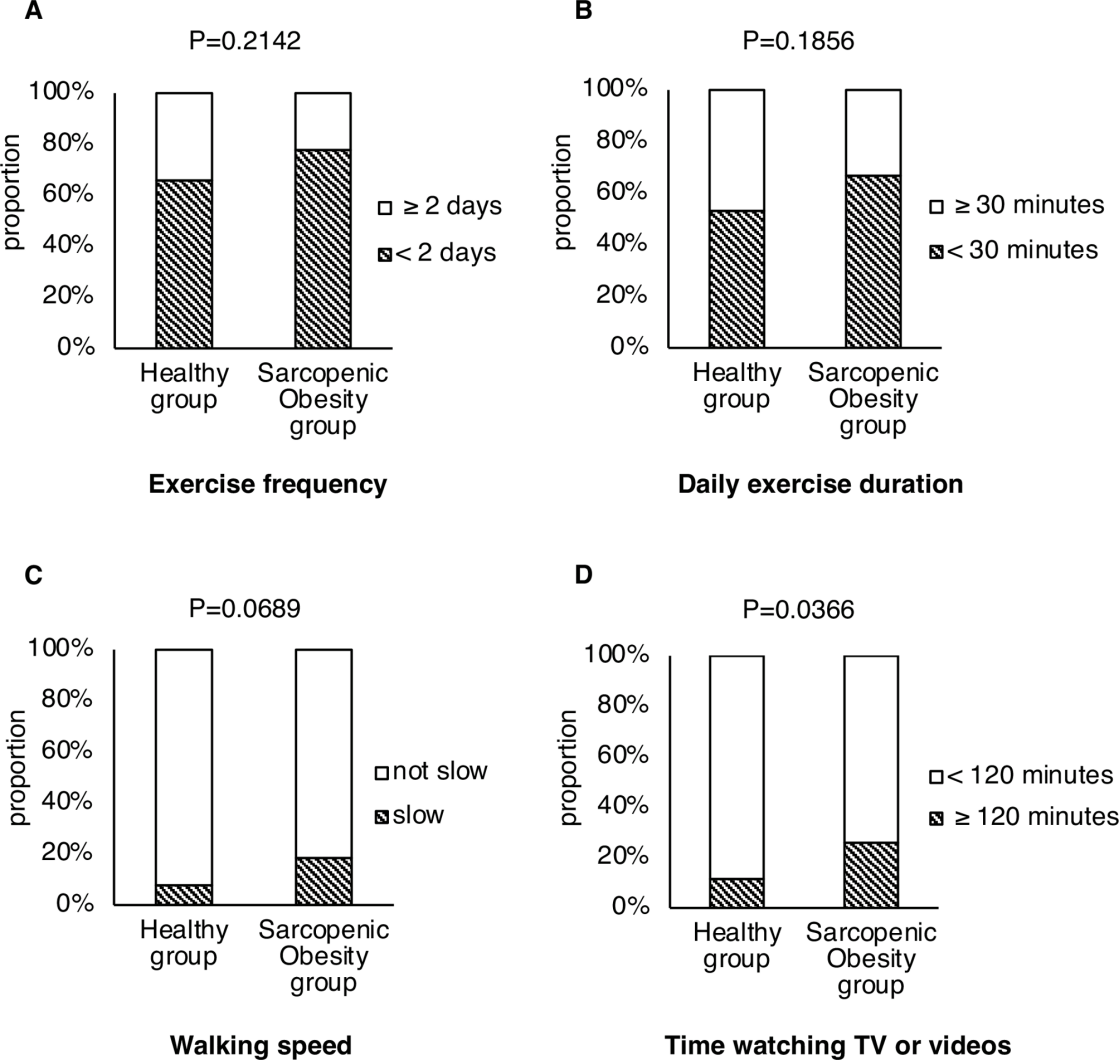
Differences in exercise-related behaviors between the healthy and sarcopenic obesity groups. Comparison of (**a**) exercise frequency, (**b**) daily exercise duration, (**c**) walking speed, and (**d**) time spent watching TV or videos between the healthy and sarcopenic obesity groups. Data are shown for participants who answered questions on exercise-related behaviors. The chi-square test was used to compare the proportions between the healthy and sarcopenic obesity groups. Healthy group: *n* = 193, sarcopenic obesity group: *n* = 27.

Exercise-related behaviors were compared between the H (*n* = 193) and SO (*n* = 27) groups (Fig. 2). The proportion of individuals who answered that their daily TV/video viewing time was greater than 120 min was higher in the SO group than in the H group (26% vs. 11%, *P* = 0.0366). Furthermore, there was a higher proportion of individuals who answered that their “walking speed is slow” in the SO group compared to the H group (19% vs. 8%, *P* = 0.0689).

### Body component analyses and correlation of clinical parameters with myostatin levels

**Fig. 3:**
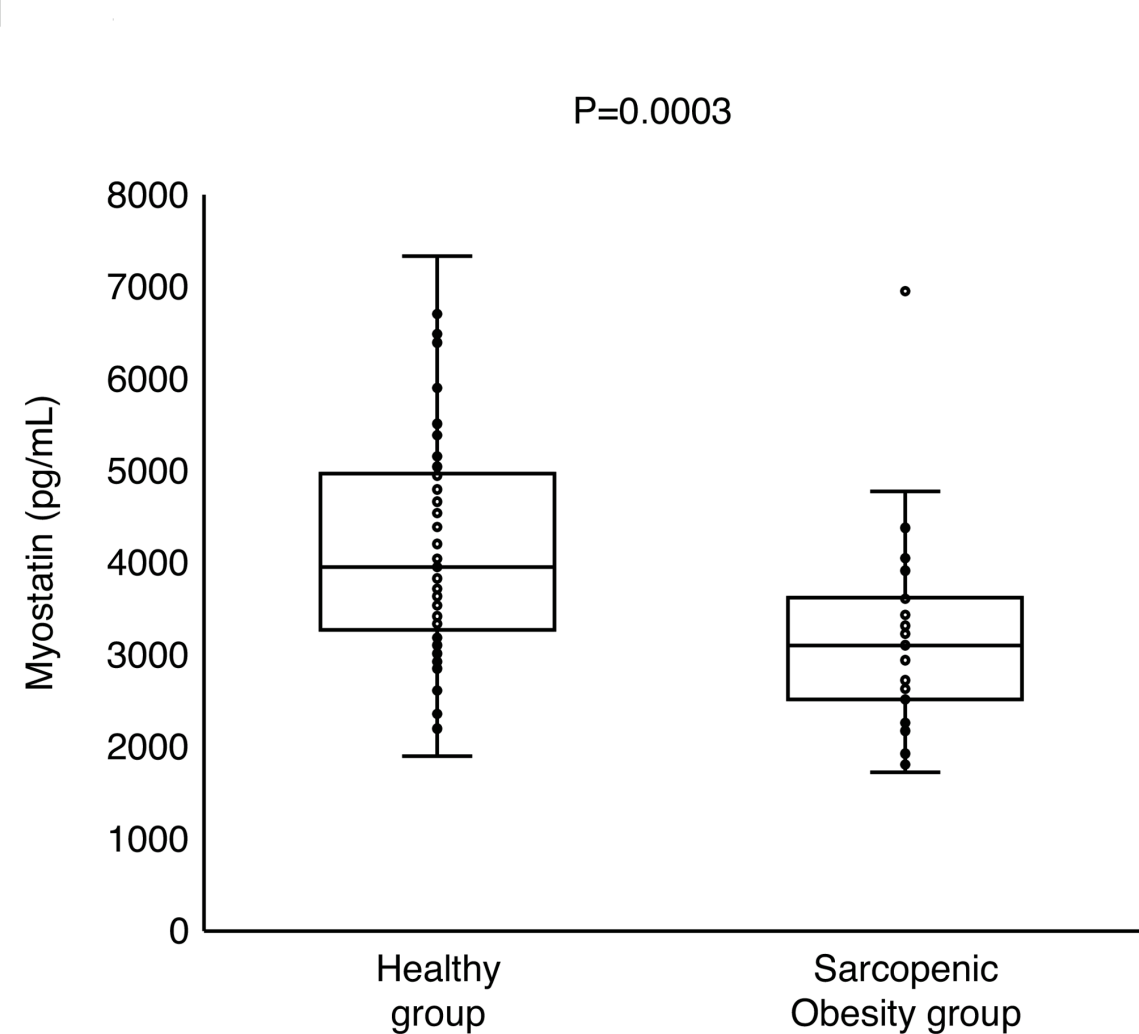
Serum myostatin levels in the healthy and sarcopenic obesity groups. Box plots show the minimum, lower quartile, median, upper quartile, and maximum myostatin levels. The Wilcoxon test was used to compare serum myostatin levels between the two groups. Healthy group: *n* = 53, sarcopenic obesity group: *n* = 27.

Myostatin and insulin levels were evaluated in 80 participants. Table 1 presents the clinical background of the participants. No significant differences in age, BMI, WC, finger-ring test results, Glu, and HbA1c levels were observed between the H and SO groups. However, the grip strength (*P* < 0.0001) was lower and the WHtR (*P* = 0.0046), ABSI (*P* = 0.0233), T-Cho (*P* = 0.0423), TG (*P* = 0.0004), LDL-C (*P* = 0.0105), insulin (*P* = 0.0236), and HOMA-R scores (*P* = 0.0262) were higher in the SO group than in the H group. Serum myostatin levels (3,107 pg/mL vs. 3,957 pg/mL, *P* = 0.0003; Fig. 3) were significantly lower in the SO group than in the H group. As the indices of skeletal muscle mass^10–12^, grip strength^10,13^, obesity^32,33^, and insulin sensitivity^32,33^ are reportedly related to circulating myostatin levels, we further assessed the differences in myostatin levels after adjusting for these factors. We found that the myostatin levels remained lower in the SO group than in the H group even after adjustment (Supplementary Table S2). Correlation analysis showed that myostatin levels significantly correlated with PBF, SMI, AST, ALT, TG, and HbA1c levels, and tended to correlate with age, Plt and Cr levels (Table 2). Among these factors, stepwise regression analysis identified SMI, HbA1c, and Plt as influencing factors of myostatin (Table 3).

**Table 1:** Clinical characteristics of study participants.

	Healthy group (*n* = 53)	Sarcopenic obesity group (*n* = 27)	*P*
Age, years	48 (45–52)	48 (44–54)	0.7869
BMI, kg/m^2^	20.6 (19.6–21.7)	20.8 (19.9–21.5)	0.9756
WC, cm	72.0 (66.8–77.8)	72.5 (70.0–76.5)	0.5278
WHtR	0.45 (0.42–0.48)	0.47 (0.45–0.50)	**0.0046**
ABSI, *10^− 3^ m^11/6^ kg^− 2/3^	75 (73–79)	78 (75–82)	**0.0233**
PBF, %	23.6 (20.7–26.6)	32.8 (31.4–34.6)	**< 0.0001**
SMI, kg/m^2^	6.3 (6.1–6.6)	5.3 (5.2–5.5)	**< 0.0001**
Finger-ring test n, (%) bigger/just fits/smaller	14/29/10 (26/55/19)	4/15/7 (*n* = 26) (15/58/27)	0.4759
Grip strength, kg	26.0 (23.5–28.8)	20.5 (18.0–22.5) (*n* = 26)	**< 0.0001**
SBP, mmHg	113 (106–123)	115 (107–124)	0.9553
DBP, mmHg	69 (64–77)	73 (67–75)	0.2963
WBC, *10^3^/µL	5.1 (4.7–6.0)	4.8 (4.3–5.8)	0.3462
Hb, g/dL	13.0 (12.2–13.4)	13.8 (12.7–14.4)	**0.0006**
Plt *10^4^, /µL	23.2 (19.3–27.2)	22.6 (20.8–26.9)	0.8787
AST, IU/L	20 (17–23)	19 (17–22)	0.2446
ALT, IU/L	13 (11–18)	12 (11–16)	0.2785
γGTP, IU/L	17 (14–24)	16 (13–22)	0.2576
BUN, mg/dL	10.9 (9.4–12.9)	11.3 (9.6–13.0)	0.7408
Cr, mg/dL	0.67 (0.62–0.74)	0.61 (0.55–0.67)	**0.0016**
UA, mg/dL	4.3 (3.8–4.9)	4.4 (3.8–5.3)	0.7097
T-Cho, mg/dL	193 (171–211)	215 (180–229)	**0.0423**
TG, mg/dL	51 (39–73)	76 (59–97)	**0.0004**
HDL-C, mg/dL	72 (60–86)	74 (55–82)	0.5756
LDL-C, mg/dL	105 (85–123)	119 (104–145)	**0.0105**
Glu, mg/dL	85 (81–90)	87 (82–90)	0.3252
HbA1c, %	5.2 (5.1–5.5)	5.2 (5.1–5.4)	0.9183
insulin, µU/mL	3.1 (2.2–4.2)	4.0 (2.9–4.6)	**0.0236**
HOMA-R	0.65 (0.45–0.87)	0.84 (0.64–1.0)	**0.0262**

Data are expressed as median (interquartile range). *BMI* body mass index, *WC* waist circumference, *WHtR* waist-to-height ratio, *ABSI* a body shape index, *PBF* percent body fat, *SMI* skeletal muscle mass index, *SBP* systolic blood pressure, *DBP* diastolic blood pressure, *WBC* white blood cell count, *Hb* hemoglobin, *Plt* platelet, *AST* aspartate aminotransferase, *ALT* alanine aminotransferase, *γGTP* γ-glutamyl transpeptidase, *BUN* blood urea nitrogen, *Cr* creatinine, *UA* uric acid, *T-Cho* total cholesterol, *TG* triglyceride, *HDL-C* high-density lipoprotein cholesterol, *LDL-C* low-density lipoprotein cholesterol, *Glu* glucose, *HbA1c* hemoglobin A1c, *HOMA-R* homeostatic Model Assessment of Insulin Resistance. The Wilcoxon test was used to compare continuous variables between the groups, and the chi-square test was used to compare the proportions of categorical values. Bold font indicates statistical significance.

**Table 2:** Correlation between serum myostatin levels and clinical characteristics.

*n* = 80	ln myostatin	
	*R*	*P*
Age	0.21	0.0617
BMI	0.079	0.4880
WC	0.021	0.8552
WHtR	0.0056	0.9605
ABSI	−0.037	0.7419
PBF	−0.28	**0.0108**
SMI	0.29	**0.0083**
Grip strength (*n* = 79)	0.058	0.6123
ln AST	0.33	**0.0032**
ln ALT	0.30	**0.0068**
ln Plt	−0.20	0.0765
ln γGTP	0.17	0.1288
ln BUN	0.13	0.2461
ln Cr	0.20	0.0823
UA	0.072	0.5270
ln T-Cho	−0.092	0.4156
ln TG	−0.24	**0.0298**
ln HDL-C	0.041	0.7203
LDL-C	−0.032	0.7807
ln Glu	0.024	0.8343
ln HbA1c	0.28	**0.0127**
ln Insulin	−0.13	0.2358
ln HOMA-R	−0.12	0.2764

*BMI* body mass index, *WC* waist circumference, *WHtR* waist-to-height ratio, *ABSI* a body shape index, *PBF* percent body fat, *SMI* skeletal muscle mass index, *AST* aspartate aminotransferase, *ALT* alanine aminotransferase, *Plt* platelet, *γGTP* γ-glutamyl transpeptidase, *BUN* blood urea nitrogen, *Cr* creatinine, *UA* uric acid, *T-Cho* total cholesterol, *TG* triglyceride, *HDL-C* high-density lipoprotein cholesterol, *LDL-C* low-density lipoprotein cholesterol, *Glu* glucose, *HbA1c* hemoglobin A1c, *HOMA-R* homeostatic Model Assessment of Insulin Resistance. Log-transformed values were expressed with *ln* at the beginning. The Pearson’s correlation coefficient analysis was used for the assessment of relationship between serum myostatin levels and clinical characteristics. Bold font indicates statistical significance.

**Table 3:** The multivariate regression analysis for myostatin.

*n* = 80	ln myostatin	
	ß	*P*
SMI	0.2792	**0.0067**
ln HbA1c	0.2362	**0.0318**
ln Plt	-0.2291	**0.0257**
ln AST	0.2024	0.0643

*SMI* skeletal muscle mass index, *HbA1c* hemoglobin A1c, *Plt* platelet, *AST* aspartate aminotransferase. Log-transformed values were expressed with *ln* at the beginning. The multiple regression analysis was performed to identify independent factors for myostatin level. Explanatory variables were determined using the stepwise method. Bold font indicates statistical significance.

## Discussion

Here, we report a prevalence of 6.3% for SO in a middle-aged Japanese female population using our specified cut-off values. As Asians exhibit a higher body fat percentage at equivalent BMI levels than Caucasians^41,42^ and African Americans^41^, numerous studies on SO have emerged, especially in Asia. However, most studies have focused on older adults and only a few reports have focused on the prevalence of SO in the middle-aged population. As SO causes metabolic disorders and vascular events^1–5^, it is important to detect SO in the relatively young population. A survey based on a Korean population reported a prevalence of 4.8% of SO in women aged 40–49 years^43^, with SO defined as SMI < 5.38 kg/m^2^, (assessed using dual-energy x-ray absorptiometry: DXA) and WC ≥ 85 cm. Another study conducted in Singapore^44^ reported that the proportion of community-dwelling women aged 21–59 years meeting criteria for both sarcopenia (SMI < 5.4 kg/m^2^ assessed by DXA and hand grip strength < 18 and/or gait speed < 1.0 m/s) and obesity (WC ≥ 80 cm or PBF > upper two quintiles, 41.4%) was 1.7% (using WC cutoff) and 1.3% (using PBF cutoff). The variability in the prevalence of SO may be due to the lack of standard criteria for SO diagnosis^40,44,45^. Therefore, to provide effective measures against SO, it is necessary to establish unified and useful diagnostic criteria. SO is often defined according to the criteria for sarcopenia and obesity determined in each study. In general, SMI using DXA or bioelectrical impedance analysis (BIA) is used to define sarcopenia, whereas BMI, abdominal circumference, and body fat percentage are used to define obesity. In this study, sarcopenia was defined according to the Asian Working Group for Sarcopenia criterion for low skeletal muscle mass, which is the most commonly used criterion in Asian populations. As our study participants were relatively young, the low physical performance criterion was omitted. Obesity was defined as PBF exceeding 30%^39,40^. Using these criteria, our study revealed that the SO group exhibited diminished grip strength and significantly elevated levels of TG, LDL-C, and HOMA-R compared with the H group. This suggests that individuals in the SO group are at risk of developing various metabolic diseases in the future, verifying the clinical validity of our SO criteria. We propose that our criteria of SMI < 5.7 kg/m^2^ and PBF ≥ 30%, measured by BIA, would be useful for diagnosing SO in the Asian female population.

While the BMI and WC in the SO group did not differ significantly from those in the H group, the WHtR and ABSI showed notable differences between the groups. This implies that these indices may serve as useful tools for identifying SO in situations where body composition analyses are not available. In addition, grip strength test results showed a significant difference between the SO and H groups in this study, which is consistent with previous reports^10,13^. As grip strength tests are simple and easy to perform, they should be incorporated into routine health checkups. Regarding exercise-related behaviors, the SO group had a higher proportion of individuals whose daily TV/video clip viewing time was greater than 120 min than the H group. Lower levels of physical activity are associated with an increased risk of SO in older adults^46,47^. Providing health guidance to encourage people to engage in physical activities might be beneficial in preventing SO.

To the best of our knowledge, no previous study has evaluated myostatin levels in apparently healthy middle-aged women. Myostatin inhibits the growth of skeletal muscles by binding to the activin type IIB myostatin receptor, activating the small mothers against decapentaplegic (Smad)-mediated pathway, and inhibiting the Akt/mammalian target of rapamycin (mTOR)/p70S6 protein synthesis pathway, which mediates differentiation in myoblasts and hypertrophy in myotubes^48,49^. Myostatin is also known to promote obesity by inhibiting fatty acid oxidation^50^ and the formation of brown adipose tissue^50,51^ which exert anti-obesity effects by increasing energy expenditure. Circulating myostatin levels are increased in individuals with sarcopenia^16–26^ and obesity^32,33^. In this context, myostatin is considered a biomarker of SO, a combination of sarcopenia and obesity. We hypothesized that serum myostatin levels might be higher in the SO group than in the H group. However, contrary to our expectations, myostatin levels were lower in the SO group than in the H group. As serum myostatin levels have been reported to be associated with skeletal muscle mass^10–12^ and obesity^32,33^, we further evaluated myostatin levels after adjusting for the relevant factors (Supplementary Table S2). Even after adjusting for these factors, myostatin levels remained lower in the SO group than in the H group. This discrepancy may be attributed to differences in the characteristics of the study participants. Previous studies have focused on the elderly and patients presenting with dysfunction of vital organs such as the liver, heart, and kidney, whereas our study focused on apparently healthy individuals with abnormalities in body composition measurements. It has been hypothesized that myostatin is appropriately suppressed in individuals with mild SO, as observed in this study; however, this feedback mechanism may be disrupted during SO progression.

Myostatin levels have been reported to positively correlate with HOMA-R, an index of insulin resistance, in obese individuals^33^. While a correlation between myostatin and HOMA-R was not observed in this study, HbA1c showed a positive correlation with serum myostatin levels. Generally, elevated HbA1c levels are caused by increased insulin resistance or decreased insulin secretion capacity. Increased serum myostatin levels are associated with type 1 diabetes and insulin secretion deficiency^52,53^. These results suggest that HbA1c might be more influenced by insulin secretory capacity than by insulin resistance in the study participants, and the positive correlation between serum myostatin and HbA1c might be explained by reduced insulin secretion capacity.

This study has some limitations. First, the small number of participants, especially those with SO, limited the statistical robustness of our findings, and may not allow them to be applied to other populations. To validate our findings, multicenter studies with larger samples are required in the future. Second, as this was a cross-sectional study, it cannot be ascertained whether the observed SO group was prone to developing metabolic disorders or cardiovascular events. Finally, the analysis did not examine factors beyond myostatin. For instance, myostatin competes with folistatin^54,55^, and other myokines, adipokines, or hepatokines^56–58^ implicated in the pathogenesis of SO were not considered in this study.

In conclusion, we revealed the prevalence of SO in middle-aged female, as defined by the criteria of SMI < 5.7 kg/m^2^ and PBF ≥ 30% measured by BIA, and found low serum myostatin levels in individuals with SO. It is important to detect SO in an early stage for prevention of metabolic disorders and cardiovascular diseases that may occur in the future. For the early detection and management of SO, body composition analyses, which are essential for the diagnosis of SO, should be widely performed, and clinically meaningful and uniform diagnostic criteria should be established. We believe that the diagnostic criteria used in this study will be useful in diagnosing SO in the general Asian female population.

## Electronic supplementary material

Below is the link to the electronic supplementary material.

Supplementary Material 1

## Data Availability

The authors confirm that the data supporting the findings of this study are available in the article.
