# Supplementary material for: Myostatin as a plausible biomarker for early stage of sarcopenic obesity
Source: Sci Rep. 2024 Nov 19;14:28629. doi: 10.1038/s41598-024-79534-5 (PMC11577097; doi:10.1038/s41598-024-79534-5)
Supplement: Supplementary file 1 — Supplementary Material 1 [file 41598_2024_79534_MOESM1_ESM.docx]

**Myostatin as a plausible biomarker for early stage of sarcopenic obesity**

Chisaki Ishibashi, MD, PhD, Kaori Nakanishi, MD, PhD^*^, Makoto Nishida, MD, PhD, Haruki Shinomiya, MD, PhD, Maki Shinzawa MD, PhD, Daisuke Kanayama MD, PhD, Ryohei Yamamoto, MD, PhD, Takashi Kudo, MD, PhD, Izumi Nagatomo, MD, PhD, and Keiko Yamauchi-Takihara, MD, PhD

**Supplementary Table S1.** Clinical characteristics of the study participants.

|  | Healthy group  (n=193) | Sarcopenia-only group (n=73) | Obesity-only group (n=139) | Sarcopenic obesity group (n=27) | *P* |
| --- | --- | --- | --- | --- | --- |
| Age, years | 47 (44–52) | 46 (40–52) | 47 (43–51) | 48 (44–54) | 0.1198 |
| BMI, kg/m^2^ | 20.6 (19.6–21.7) | 18.6 (17.6–19.5)^a,c^ | 24.6 (22.8–26.9)^a,b^ | 20.8 (19.9–21.5)^b,c^ | **<0.0001** |
| WC, cm | 71.5 (68.0–76.5) | 66.0 (64.0–69.5)^a,c^ | 81.0 (76.5–88.5)^a,b^ | 72.5 (70.0–76.5)^b,c^ | **<0.0001** |
| WHtR | 0.44 (0.43–0.47) | 0.42 (0.41–0.45)^a,c^ | 0.51 (0.48–0.55)^a,b^ | 0.47 (0.45–0.50)^a,b,c^ | **<0.0001** |
| ABSI, *10^-3^m^11/6^kg^-2/3^ | 76 (73–79) | 76 (74–79) | 77 (74–80) | 78 (75–82) | **0.0489** |
| PBF, % | 24.4 (21.0–27.4) | 24.1 (21.3–26.6)^c^ | 34.7 (32.4–38.2)^a,b^ | 32.8 (31.4–34.6)^a,b,c^ | **<0.0001** |
| SMI, kg/m^2^ | 6.2 (6.0–6.5) | 5.4 (5.2–5.6)^a,c^ | 6.5 (6.1–6.9)^a,b^ | 5.3 (5.2–5.5)^a,c^ | **<0.0001** |
| Finger-ring test, n (%)  bigger/just fits/smaller | 69/80/44  (36/41/23) | 5/31/37  (7/42/51) | 96/37/6  (69/27/4) | 4/15/7  (15/58/27)  (n=26) | **<0.0001** |
| Grip strength, kg | 26.0 (23.5–28.5)  (n=191) | 22.5 (20.5–25.4)^a,c^  (n=72) | 25.0 (21.5–28.5)^b^ | 20.5 (18.0–22.5)^a,b,c^  (n=26) | **<0.0001** |
| SBP, mmHg | 113 (105–122) | 108 (100–119)^a,c^ | 120 (110–131)^a,b^ | 115 (107–124) | **<0.0001** |
| DBP, mmHg | 69 (61–75) | 65 (61–70)^a,c^ | 73 (66–82)^a,b^ | 73 (67–75)^b^ | **<0.0001** |
| WBC, *10^3^/μL | 5.0 (4.4–5.8) | 4.9 (4.3–5.5)^c^ | 5.7 (4.8–6.6)^a,b^ | 4.8 (4.3–5.8) | **<0.0001** |
| Hb, g/dL | 12.9 (12.3–13.5) | 13.4 (12.6–13.7) | 13.2 (12.5–14.0)^a^ | 13.8 (12.7–14.4)^a^ | **0.0008** |
| Plt *10^4^, /μL | 23.1 (20.2–26.7) | 23.8 (19.8–27.0)^c^ | 26.0 (22.5–29.6)^a,b^ | 22.6 (20.8–26.9)^c^ | **<0.0001** |
| AST, IU/L | 20 (17–23) | 20 (17–23) | 19 (16–22) | 19 (17–22) | 0.2275 |
| ALT, IU/L | 13 (11–18) | 14 (11–17) | 15 (11–20) | 12 (11–16) | 0.0683 |
| γGTP, IU/L | 17 (13–23) | 17 (13–22) | 16 (13–24) | 16 (13–22) | 0.7681 |
| BUN, mg/dL | 11.6 (9.9–13.7) | 11.5 (9.7–13.1) | 10.7 (9.1–13.2) | 11.3 (9.6–13.0) | 0.1000 |
| Cr, mg/dL | 0.67 (0.61–0.73) | 0.62 (0.57–0.66)^a^ | 0.63 (0.57–0.71)^a^ | 0.61 (0.55–0.67)^a^ | **<0.0001** |
| UA, mg/dL | 4.2 (3.7–4.9) | 4.2 (3.6–4.8)^c^ | 4.7 (3.9–5.4)^a,b^ | 4.4 (3.8–5.3) | **0.0011** |
| T-Cho, mg/dL | 194 (174–218) | 197 (171–222) | 202 (183–226) | 215 (180–229) | 0.0770 |
| TG, mg/dL | 51 (42–73) | 47 (39–74)^c^ | 77 (58–102)^a,b^ | 76 (59–97)^a,b^ | **<0.0001** |
| HDL-C, mg/dL | 74 (63–84) | 72 (66–84)^c^ | 64 (55–72)^a,b^ | 74 (55–82) | **<0.0001** |
| LDL-C, mg/dL | 105 (90–126) | 106 (91–125)^c^ | 120 (101–143)^a,b^ | 119 (104–145)^a^ | **<0.0001** |
| Glu, mg/dL | 85 (81–89) | 82 (79–90)^c^ | 89 (85–94)^a,b^ | 87 (82–90) | **<0.0001** |
| HbA1c, % | 5.2 (5.1–5.5) | 5.2 (5.1–5.4)^c^ | 5.3 (5.2–5.6)^a,b^ | 5.2 (5.1–5.4) | **0.0035** |

Data are expressed as the median (interquartile range). P values were used for comparisons among the four groups. Bold font indicates statistical significance. For continuous variables, a post hoc Steel-Dwass test was performed: ^a^ *P*<0.05 vs. healthy group; ^b^ *P*<0.05 vs. sarcopenia-only group; ^c^ *P*<0.05 vs. obesity-only group. *BMI* body mass index, *WC* waist circumference, *WHtR* waist-to-height ratio, *ABSI* a body shape index, *PBF* percent body fat, *SMI* skeletal muscle mass index, *SBP* systolic blood pressure, *DBP* diastolic blood pressure, *WBC* white blood cell count, *Hb* hemoglobin, *Plt* platelet, *AST* aspartate aminotransferase, *ALT* alanine aminotransferase, *γGTP* γ-glutamyl transpeptidase, *BUN* blood urea nitrogen, *Cr* creatinine, *UA* uric acid, *T-Cho* total cholesterol, *TG* triglyceride, *HDL-C* high-density lipoprotein cholesterol, *LDL-C* low-density lipoprotein cholesterol, *Glu* glucose, *HbA1c* hemoglobin A1c, *HOMA-R* homeostatic Model Assessment of Insulin Resistance.

In the sarcopenic obesity group, the BMI, WC, WHtR, PBF, grip strength, DBP, and TG levels were significantly different from those in the sarcopenia-only group, whereas the BMI, WC, WHtR, PBF, SMI, grip strength, and Plt levels were significantly different from those in the obesity-only group.

**Supplementary Table S2.** Comparison of myostatin levels between the healthy and sarcopenic obesity groups using analysis of covariance (ANCOVA).

| N=80 | Healthy group  (n=53) | Sarcopenic obesity group (n=27) | P value for groups |
| --- | --- | --- | --- |
| Myostatin |  |  |  |
| Model 1 | 3,958 (3,633–4,311) | 3,092 (2,677–3,573) | **0.0045** |
| Model 2 | 4,188 (3,831–4,578) | 3,196 (2,662–3,838) | **0.0099** |
| Model 3 | 4,013 (3,686–4,368) | 3,002 (2,627–3,431) | **0.0005** |
| Model 4 | 3,940 (3,624–4,283) | 2,938 (2,595–3,327) | **0.0002** |

Data are expressed as estimated marginal means (95% CI). Bold font indicates statistical significance. Model 1: adjusted for log-transformed Cr level;

Model 2: adjusted for grip power;

Model 3: adjusted for WHtR;

Model 4: adjusted for log-transformed insulin level;

*Cr* creatinine, *SMI* skeletal muscle mass index, *PBF* percent body fat*, WHtR* waist-to-height ratio.

The ANCOVA was conducted to determine the difference of myostatin levels between the healthy group and the sarcopenic group. As the indices of skeletal muscle mass, grip strength, obesity, and insulin sensitivity are reportedly related to circulating myostatin levels, we assessed the differences in myostatin levels after adjusting for Cr level, grip power, WHtR, and insulin level. As myostatin, Cr, and insulin levels were non-normally distributed, these variables were log-transformed in the test. The test revealed that serum myostatin levels remained lower in the SO group than in the H group after adjustment for Cr level, grip power, WHtR, and insulin level.
